# Effects of the *Empowering Coaching*™ training program on the coach created motivational climate, psychological need satisfaction and team cohesion

**DOI:** 10.3389/fpsyg.2026.1566111

**Published:** 2026-08-19

**Authors:** Sofia Mosqueda, Jeanette M. López-Walle, Luis Ródenas Cuenca, José Tristán, Inés Tomás, Isabel Balaguer, Joan L. Duda

**Affiliations:** 1 Facultad de Organización Deportiva, Universidad Autónoma de Nuevo León, San Nicolás de los Garza, Nuevo León, Mexico; 2 Department of Methodology, Faculty of Psychology and Speech Therapy, University of Valencia, Valencia, Spain; 3 Department of Social Psychology, Faculty of Psychology and Speech Therapy, University of Valencia, Valencia, Spain; 4 School of Sport, Exercise & Rehabilitation Sciences, College of Life and Environmental Sciences, University of Birmingham, Birmingham, United Kingdom

**Keywords:** motivational climate, basic psychological needs, cohesion, empowering coaching, coach education

## Abstract

**Introduction:**

This study examined the longitudinal effects of a coach education program (based on *Empowering Coaching*™), implemented in a school-based setting across the sport season, on young athletes’ perceptions of the motivational climate, satisfaction of basic psychological needs, and team cohesion across a sport season.

**Methods:**

Participants were 314 athletes from Mexico (12–16 years, *M* = 15.11, *SD* = 1.30) at baseline, of whom 143 completed post-intervention assessments (45.5% retention). Twenty-five coaches engaged in workshops and guided reflections over a 7-month period. Athletes completed questionnaires assessing the prevailing motivational climate, need satisfaction, and cohesion at the beginning and end of the season.

**Results:**

Paired-sample analyses revealed significant small-to-moderate improvements in athletes’ perceptions of an empowering climate, autonomy and relatedness satisfaction, and social cohesion, whereas competence satisfaction and task cohesion showed no significant change. To further examine hypothesized mechanisms, structural equation modeling indicated that perceptions of an empowering climate directly predicted changes in task cohesion and indirectly predicted social cohesion through changes in autonomy satisfaction.

**Discussion:**

The findings highlight the potential of an extended, collaborative and multi-activity delivery of *Empowering Coaching*™ training to promote young athletes’ feelings of autonomy and belonging as well as group functioning within competitive school sport.

## 1 Introduction

Coaches are significant figures in the sport context and can influence the quality of athletes’ participation, both positively and negatively (Duda and Balaguer, 2007; Smoll and Smith, 2006). The interactions between coaches and their athletes shape not only individual outcomes (such as competence perceptions) but also team-level processes and outcomes (such as cohesion; Spink, 2020).

Applied to the sport domain, contemporary motivation theories - such as achievement goal theory (AGT; Ames, 1992; Nicholls, 1989), and self-determination theory (SDT; Deci and Ryan, 1985, 2000)—have paid particular attention to the influence of significant others (e.g., the coach) in shaping a psychological climate that is a central determinant of variations in athlete and team functioning. This climate encompasses the coach’s behaviors; what the coach does, says and the way he or she structures the training and competitive environment (Duda, 2001). Variability in the perceived motivational climate—the social environment created through coaches’ behaviors, communication, and leadership style—has been shown to elicit adaptive as well as maladaptive responses (Keegan et al., 2014; Fabra et al., 2021).

Achievement Goal Theory (AGT; Ames, 1992; Nicholls, 1989) places emphasis on the task-involving (mastery-oriented) and ego-involving (performance-oriented) aspects and their implications for how athletes define success and judge their competence as well as ensuing outcomes (Duda and Balaguer, 2007). Extensive sport research has pointed out the importance of promoting task-involving motivational climates in sport to enhance more adaptive outcomes (such as positive affect and satisfaction) in both grassroots and elite sport (Lochbaum and Sisneros, 2024).

According to SDT (Deci and Ryan, 2000; Ryan and Deci, 2017), optimal functioning arises when the social environment nurtures the satisfaction of the three Basic Psychological Needs: autonomy (feeling volitional and agentic), competence (feeling effective), and relatedness (feeling connected to others). Related to the social environment, SDT emphasizes autonomy support, social support or involvement and controlling behaviors, and their implications for need satisfaction (as well as need frustration). A recent systematic review supported positive associations between coaches’ autonomy support, need satisfaction, and indicators of athletes’ wellbeing. These effects emerged, regardless of the culture or type of sport analyzed (Mossman et al., 2022).

An empowering and disempowering motivational climate model has been proposed which incorporates the dimensions and predictions emanating from the two previous theories (Duda, 2013; Duda et al., 2024). This framework describes the motivational climate according to the extent to which it is more or less empowering and disempowering. An empowering motivational climate is marked by strong task-involving, autonomy-supportive, and socially supportive behaviors, whereas a disempowering climate is characterized by pronounced ego-involving and controlling behaviors (Duda, 2013; Duda and Appleton, 2016; Duda et al., 2024). A scoping review revealed that empowering climates created by coaches are associated with athletes’ optimal functioning and greater intentions to continue participating in sports (Birr et al., 2023).

### 1.1 The motivational climate and team cohesion

Existing research on the concomitants of the motivational climate in sport has tended to focus on the prediction of individual (athlete) processes and outcomes. Not surprising, the prevailing motivational climate in sport also holds implications for how teams are functioning. One of the central processes in the dynamics of sports teams is cohesion, defined as the “tendency for a group to stick together and remain united in the pursuit of its instrumental objectives and/or for the satisfaction of member affective needs” (Carron et al., 1998, p. 213). This literature suggests there are two dimensions of cohesion, namely social cohesion (team members trust and enjoy each other’s company) and task cohesion (team members share goals, strategies and understand their role(s) on the team).

According to Eys and Brawley (2018), although cohesion does not always lead to better performance, cohesive teams tend to exhibit more optimal functioning, higher quality team dynamics and realize positive outcomes. It was likely the awareness of these positive effects (Spink, 2020) that led researchers to analyze the antecedents of cohesion. One of these is the motivational climate created by significant others, in particular the behaviors and leadership style of the coach. Research analyzing the relationship between the motivational climate created by coaches and cohesion has most frequently been based on achievement goal theory. The results obtained in cross-sectional and longitudinal (e.g., Balaguer et al., 2015; Eys et al., 2013; Heuzé et al., 2006; McLaren et al., 2015) studies have revealed positive relationships between perceptions of a task-involving coach-created motivational climate and reported task and social cohesion on sport teams.

Few studies have examined the relationships between a motivational climate, as characterized via a self-determination theory lens, and cohesion. One notable contribution is research by Moreno-Murcia et al. (2020). They analyzed how teacher autonomy support is related to group cohesion among university students in Spain, Portugal, Brazil, Chile, and Mexico. Findings revealed teacher autonomy support to be positively associated with need satisfaction, which in turn was linked to intrinsic motivation, ultimately predicting higher levels of group cohesion. These pathways were found to be consistent across the five Ibero-American countries studied (Moreno-Murcia et al., 2020).

Research examining the relationships between perceptions of empowering and disempowering motivational climates and team cohesion is particularly limited. To our knowledge, one (unpublished) study, involving a small sample of rhythmic gymnasts from the Czech Republic has addressed this question (Laroëre et al., 2026). The researchers found ‘empowered’ (in terms of their perceptions of the motivational climate) athletes to report higher team cohesion than those participating in a disempowering climate. Examining the effects of a more empowering motivational climate on task and social cohesion in young athletes is one objective of the present research.

### 1.2 The mediational role of basic psychological needs

Self-determination theory (Deci and Ryan, 2000) posits that optimal functioning and development depend on social environments that support individuals’ basic psychological needs. These needs—competence (feeling effective in one’s activities), autonomy (experiencing oneself as the origin of one’s actions), and relatedness (feeling connected to and accepted by significant others)—are considered innate and universal foundations for psychological growth and optimal functioning (Deci and Ryan, 2000; Ryan and Deci, 2000). Conversely, when these needs are not satisfied or are actively thwarted, self-determination theory predicts increased ill-being and maladjustment (Ryan and Deci, 2000). More specifically, Basic Psychological Needs Theory (Deci and Ryan, 2000), a sub-theory of SDT, assumes that the psychological needs mediate the influence(s) of the motivational climate on outcomes.

Past research has found perceptions of a task-involving motivational climate to positively predict basic psychological need satisfaction (BPNS) in athletes (e.g., Álvarez et al., 2012; Reinboth and Duda, 2006; Valero-Valenzuela et al., 2024; Zheng et al., 2023). Consonant with the predictions of SDT, need supportive features of the coach-created climate (such as autonomy support) relate to greater athlete BPNS in cross-sectional (Adie et al., 2008) and longitudinal studies (Balaguer et al., 2012).

Grounded in Duda’s (2013) theoretically integrated model of the motivational climate, a study conducted by Castillo-Jiménez et al. (2017) involving young male soccer players found that an empowering climate was a positive predictor of basic psychological need (BPN) satisfaction. Similarly, Ramírez (2020) reported that a perceived empowering climate was positively associated with BPN satisfaction, whereas a disempowering climate was positively related to BPN thwarting. Comparable results were reported by Chu et al. (2020) in their study of student-athletes.

### 1.3 Needs satisfaction and team cohesion

The satisfaction of the basic psychological needs is assumed to be fundamental to individual and collective thriving (Ryan and Deci, 2017). Several studies have shown that satisfaction of basic psychological needs is associated with higher levels of group cohesion (e.g., Moreno-Murcia et al., 2020). Nascimento et al. (2017), in a study with male futsal players, found basic psychological need satisfaction to positively predict both task and social cohesion. In another study Nascimento et al. (2019) found that the satisfaction of all three needs (autonomy, competence, and relatedness) was positively associated with task cohesion, whereas only satisfaction of relatedness and competence was associated with social cohesion. Heuzé et al. (2018) reported that task cohesion was positively related to satisfaction of the need for relatedness. Greater perceived social cohesion was associated with higher levels of autonomy and relatedness satisfaction, but with lower levels of competence satisfaction.

The present study also considers BPNS in regard to the prediction of team cohesion. In particular, we will be testing the mediational role of BPNS in the relationship of an empowering motivational climate to task and social cohesion.

### 1.4 Promoting more adaptive motivational climates

There is a wealth of research supporting the role of the coach-created motivational climate in regard to variability in athlete and team functioning (e.g., Duda and Balaguer, 2007; Harwood and Thrower, 2020). Such evidence lays the bases for developing theoretically grounded coach education programs so that more adaptive motivational climates are advanced and optimal engagement in athletes and teams is promoted (see Langan et al., 2013). Some existing theory-based interventions, with these aims, pull from AGT, exemplified by the Mastery Approach to Coaching (MAC; Smith et al., 2007), while others are grounded in SDT (e.g., Cheon et al., 2015; Reynders et al., 2019).

A motivational climate intervention exists which is rooted in the empowering and disempowering framework (Duda, 2013) and incorporates constructs and principles from AGT and SDT as well as techniques related to behavior change. The *Empowering Coaching*™ training program, which has at is theoretical core this integrated framework, is designed to foster environments that concurrently support all three needs by promoting task-involving behaviors (which facilitate competence), autonomy-supportive interactions, and socially supportive relationships (Duda, 2013; Duda et al., 2018, 2024). This integrated approach aligns with SDT’s core premise that these needs are functionally interrelated and should not be treated in isolation.

The *Empowering Coaching*™ program aims to equip coaches with an in-depth understanding of motivational processes and their downstream effects. Specifically, the workshop entails working with coaches so that they can recognize how the design of practice sessions, the delivery of performance feedback, and the communication of evaluative standards can significantly influence athletes’ basic psychological need satisfaction, motivation quality, and indicators of their wellbeing and functioning. With awareness of the key concepts and furthered understanding, participating coaches are encouraged to develop strategies which would be more empowering and reflect on behaviors (and consider modifying or eliminating) which can be disempowering for their athletes. Fundamental to the training is that the coaches participating co-develop their approaches to be more empowering and less disempowering in exchange with the workshop leaders and importantly, in discussion with other coaches (Balaguer et al., 2021).

A limitation in the current motivational climate-centered literature is that most interventions focus on improving individual-level outcomes (e.g., motivation, enjoyment, dropout intentions) with less attention to the effects of the program on social or group-level processes and outcomes, such as team cohesion. One notable exception is the work of McLaren et al. (2015). In a study that was AGT-grounded, an adapted version of Smoll and Smith’s Mastery Approach to Coaching content was delivered to youth soccer coaches from several recreational clubs. Trained coaches were found to exhibit more task-involving behaviors and their players reported increased team and social cohesion by the end of the season.

The large majority of youth athletes train and compete within teams. Besides clubs and community programs, many young people engage in team sports in school sport programs. Understanding how empowering climates influence group dynamics would be particularly important in school sport settings, where social connections often drive continued participation (Coleman et al., 2021; Weiss et al., 2021).

Although the theoretical backgrounds to creating more adaptive motivational climates is well established, less is known about the practical challenges coaches face when applying such approaches in everyday practice. Time constraints, competing demands (e.g., academic responsibilities in school settings), and limited access to professional development opportunities may limit the adoption of empowering strategies (Smoll and Smith, 2006). In Latin America, these challenges are often compounded by resource limitations and institutional barriers. Few if any studies have systematically examined the implementation of theoretically-grounded coach education programs, which are focused on promoting empowering climates, in school-based sport programs in a Latin American country.

In terms of effectively applying the content addressed in training programs to their practice, the often seen one-off workshop and didactic approaches to coach education has been criticized. The former can lead to a lack of recalling of what was addressed in the training; the latter encourages passive learning. It has been suggested that formal coach education would be more effective if the coach and the coach educator have opportunities to work together (Vella et al., 2013). Further, the value of coach learning being supported over time has been advocated (Allen et al., 2025) including involving the coaches in a variety of learning experiences (Mesquita et al., 2014). As well, the coach education (coach developer) literature points to the positive implications of practical observations in coach learning (Cushion, 2018). The present study considers these gaps and drawbacks by implementing and testing the effects of a collaborative, extended and multi-activity version of *Empowering Coaching*™ training in a Mexican school sport context that is delivered over the course of a season.

Drawing from Duda’s (Duda, 2013; Duda and Appleton, 2016) model of empowering and disempowering climates, one key aim of this study was to examine the effects of the training. That is, we were interested in whether the young athletes’ views about how empowering the coach-created motivational climate is, their need satisfaction, and how cohesive their team is (in regard to task and social aspects) changed over the course of the season/before and after their coaches engaging in an extended *Empowering Coaching™* training program. It was predicted that (a) the perceived empowering features of the coach-created motivational climate, (b) reported need satisfaction (autonomy, competence, and relatedness), and (c) reported task and social cohesion will increase from the baseline (pre-training) to the end of season (completion of training) assessment.

Drawing from existing literature and theoretical predictions, we also aimed to test a mediation model assuming changes in task cohesion and social cohesion will covary with changes in the hypothesized antecedents, namely, players’ perceptions of an empowering motivational climate and the degree to which players perceive their basic psychological needs to be satisfied. The model also assumed that psychological needs satisfaction (autonomy, competence, and relatedness) will mediate relationships between the perceptions of the empowering climate and task cohesion and social cohesion. Finally, the model also considered direct effects between changes in an empowering climate to task cohesion and social cohesion.

**Figure 1:**
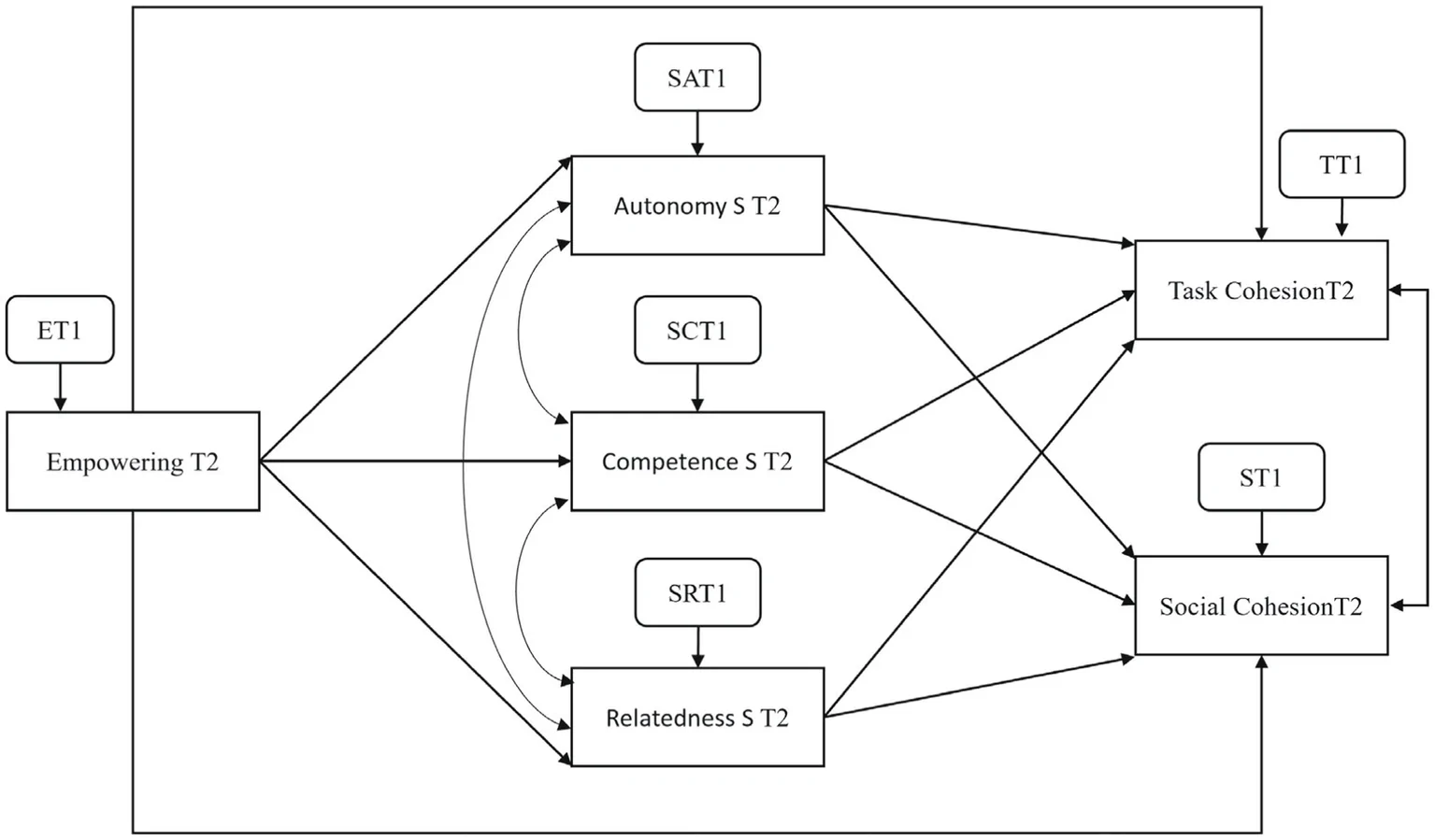
The hypothesized process model. ET1 = empowering climate time 1, SAT1 = autonomy satisfaction on time 1, SCT1 = competence satisfaction on time 1, SRTI = relatedness satisfaction on time 1, TT1 = task cohesion on time 1, ST1 = social cohesion on time 1, T2 = Time 2, S = satisfaction.

More specifically, in regard to the hypothesized process model (Figure 1), we predicted:

1. Changes in the players’ perceptions of empowering climate would positively predict changes in basic psychological need satisfaction (autonomy, competence, and relatedness).
2. Increases in psychological need satisfaction (autonomy, competence, and relatedness) will positively predict changes in task cohesion and social cohesion.
3. The relationship between changes in the perceptions of the empowering climate and task and social cohesion will be mediated by changes in psychological need satisfaction (autonomy, competence, and relatedness).
4. Changes in the perceptions of the empowering climate will also directly and positively predict changes in task cohesion and social cohesion.

## 2 Methods

### 2.1 Design

The study employed a quasi-experimental, non-randomized, time-lagged design with convenience sampling (Ato et al., 2013). Two assessments were conducted to capture within-subject change: one prior to the intervention (T1) and one mid-year (T2). While this design allowed the examination of change over time, it is important to note that the absence of a control or comparison group limits internal validity and restricts causal inference, as changes observed may also reflect external influences.

### 2.2 Sample and procedure

This study entailed the delivery of an extended version of *Empowering Coaching™* training to 25 (22 male, 3 female) Mexican coaches of 17 sport teams (with soccer and basketball being the two major sports) at a private elementary school (6th to 9th grade). The perceptions of the young athletes coached (*n* = 314), who were between 12 and 16 years old (*M* = 15.11, *SD* = 1.30 years), were assessed at two time points via questionnaires assessing the targeted variables (perception of the empowering and disempowering climate, the satisfaction of the need for autonomy, competence and relatedness, and team cohesion). The Time 1 (T1) assessment took place 1 month after the start of the season (September), to ensure that the young athletes had enough time to form perceptions of the climate created by their coaches and degree of cohesion on their teams. This served as a baseline assessment occurring before their coaches undertook the *Empowering Coaching™* training delivered over 7 months. The T2 assessment took place in March of the academic year.

Initially, multiple schools were contacted and invited to participate in the study. Importantly, there was no formal inclusion criterion regarding the organizational structure of the schools. The only established requirement was that the school authorities formally authorized both the implementation of the intervention and the collection of research data (e.g., questionnaires, video recordings). However, during the recruitment phase, most schools declined participation after reviewing the project details. The primary reasons cited by school directors were the extended duration of the intervention and the time commitment required from coaches (e.g., attendance at workshops, availability for session recordings). As a result, only one private school agreed to participate under these conditions.

As a result, participant recruitment was determined by school affiliation, with all eligible student-athletes enrolled in the targeted sports programs at the participating institution included in the study. One hundred and forty-three of the initial sample of young athletes also completed the questionnaire pack at time two (*M* = 13.19, *SD* = 1.09). Only the athletes with responses at both T1 and T2 were used in the analyses of this study. To test possible differences between those athletes who were present only at T1 and those who took part in both assessment periods, we conducted a one-way multivariate analysis of variance (MANOVA) with athletes’ perceptions of the coach created empowering climate, satisfaction of the needs for autonomy, competence and relatedness, and team cohesion (task and social) as the dependent variables. No significant differences emerged (*F*_(6,450)_ = 2.09, *p* = 0.06). Additionally, we compared both groups on gender, age and type of sport. Results of chi-square tests indicated that there were no differences on gender (*χ^2^* (1) = 1.92, *p* > 0.05), and type of sport (*χ^2^* (4) = 3.19, *p* > 0.05). However, results of a *t*-test indicated that respondents (mean age = 15.19) differed from non-respondents (mean age = 13.19) on age (*t* (304) = 15.89, *p* < 0.001).

Briefly, A total of 314 adolescent athletes from 17 sports teams participated in the baseline data collection (T1) in September. At the follow-up assessment (T2) in March, 143 athletes completed the post-intervention questionnaire, representing a retention rate of 45.5%. However, as the attrition analysis (MANOVA) indicates, there was no significant difference with respect to the study variables or the sociodemographic variables, except for age with the initial group (*n* = 314) showing higher values on age.

The reasons for participant attrition between T1 and T2 could not be systematically tracked, as the data collection process did not allow for individualized follow-up on athletes who discontinued participation. Therefore, we cannot provide specific explanations (e.g., withdrawal from team, absences, school transfer) for the loss in sample size. Only those athletes who completed assessments at both time points (*n* = 143) were included in the final analysis.

Regarding power analysis and considering the sample size of the study (*n* = 143), with this sample we should have enough statistical power to detect relevant relationships between the model variables. According to sample size and statistical power calculations in multiple regressions, assuming a small effect size (*f^2^* = 0.11) for a maximum number of predictors (8) and an alpha level of 0.05, in order to attain a statistical power level of 0.80, the required sample size would be 145 (Faul et al., 2009). As indicated, the study sample was composed of 143 students, thus, we were 2 athletes shy of the required sample size to attain an adequate power level.

With respect to the process informed consent was first obtained from the coaches and directors of the private school, as well as from the legal guardians of the student athletes. The student athletes then consented to be involved after they were informed of the overall purpose of the study, assured of the anonymity of their responses and that they had the freedom to withdraw at any time without negative consequence. The study protocol was reviewed and approved by a university-affiliated ethics board. We then proceeded to the first data collection (T1, at the start of the school year) during which athletes responded to the different scales tapping the targeted variables.

At both time points, athletes completed standardized questionnaires assessing perceptions of the empowering motivational climate, satisfaction of the three basic psychological needs, and task and social cohesion. Two control variables were considered: type of sport (included as two dummy variables to represent the three groups: basketball, soccer, and others) and gender (male and female).

### 2.3 Intervention components

An intervention program, based on the *Empowering Coaching™* content, methodology was implemented over the course of 7 months (September–March of following year). Throughout this time period, coaches were instructed on the theoretical framework, concepts and principles behind what constitutes empowering motivational climates. Additionally, they were also taught practical strategies (and suggest strategies on their own) for promoting a more empowering climate through workshops delivered by trained personnel. The coaches also engaged in self-reflections and other activities via small group discussions and workbooks which summarized workshop content. The coaches were filmed while delivering a training session at the beginning and at the end of the season and these were reviewed. More specific aspects of the extended training program are described below:

Two workshops of 4 h each were held, given by two previously trained researchers and members of the research team: Workshop 1 “Foundations of *Empowering Coaching™*” had as its main objective to teach the theoretical bases behind the what, why and how of an empowering climate. Workshop 2 “Introducing the CLIMATE strategies” (Cooperative contribution, Learning emphasized, Intrinsic focus, Mastery oriented, Authority with autonomy, Taking others’ perspective, Evaluation of effort and improvement) within the *Empowering Coaching™* training entailed explaining these strategy approaches and providing practical examples that were specific to each sport. Coaches also provided examples of each of the CLIMATE strategies in regard to exchanges with their athletes and team, with the aim to promote motivational climates that are more empowering for individual athletes and the team as a collective.

Approximately 5 weeks later, the coaches were divided by sport, and two follow-up workshops were held: “Remembering Concepts from Workshop 1” where, in addition to reviewing the theoretical principles and concepts embedded in *Empowering Coaching™,* self-reflections were made in regard to the behaviors and strategies captured in the initial filmed session. In the second workshop, “Remembering concepts from the second workshop,” the CLIMATE strategies were reviewed and reinforced through the writing by the coaches of three sessions plans in which coaches indicated the empowering strategies they were going to implement: at least three strategies per session were requested, one strategy in the initial phase (warm-up), another different strategy in the central phase (main activity), and a third strategy in the closing phase (cool-down). The session plans were evaluated, promoting personal reflection in the trainer on “what he says,” “what he does” and “why” in creating empowering climates.

These plans and the filming were then used by the tutors in an approximately 2 h “Individual Focus” consultation. During each consultation, a member of the research team worked with each coach individually, securing their views regarding strategies employed and giving personalized feedback regarding the last observation of their coaching. All coaches completed all workshops; however, no formal adherence metric (e.g., session fidelity checklist or observational scoring) was systematically applied, which is acknowledged as a limitation of the study.

Once the training program was completed, the second application of the evaluation instruments was carried out (March).

To enhance transparency and theoretical clarity while respecting intellectual property protections, we present a logic model (see Table 1) that outlines the key components of the *Empowering Coaching*™ intervention, its theoretical foundations, the behavioral mechanisms targeted during implementation, and the expected short-term outcomes. This framework illustrates how the intervention is designed to promote an empowering motivational climate aligned with both AGT and SDT principles.

**Table 1:** Logic model of the *Empowering Coaching™*.

Intervention components	Theoretical foundations	Proximal mechanisms (targets)	Expected short-term outcomes
Task-involving strategies (e.g., effort focus, self-referenced goals)	Achievement Goal Theory (AGT)	Athlete perceptions of task climate	Increased task orientation
Autonomy-supportive strategies (e.g., meaningful choices, rationale)	Self-Determination Theory (SDT)	Basic psychological need satisfaction (autonomy, competence, relatedness)	Increased need satisfaction
Social support behaviors (e.g., empathy, inclusive communication)	Self-Determination Theory (SDT)	Athlete perception of being in related environments and with a role in the team	Greater athlete enjoyment and engagement
Coach education (workshops, guided reflection, observation feedback)	AGT + SDT	Coach awareness and application of empowering behaviors	Improved coach–athlete relationships

Although the intervention duration was sufficient to allow the consolidation of training content, a notable limitation is that no formal adherence or fidelity monitoring system was applied. For example, neither observational scoring rubrics nor session checklists were implemented to systematically document whether coaches consistently applied the strategies taught. This lack of fidelity tracking restricts conclusions about the effects of the “dose” of the intervention actually delivered and the consistency across coaches.

### 2.4 Instruments

To measure the young athletes’ perceptions of the degree to which their coach created an empowering motivational climate, we used the 17 items Empowering scale from the Mexican version (Castillo-Jiménez et al., 2017; Mosqueda et al., 2019) of the Empowering and Disempowering Motivational Climate Questionnaire-Coach (EDMCQ-C; Appleton et al., 2016), which contains items tapping task-involving coach behaviors (e.g., “My coach encourages players to try new skills”), autonomy supportive strategies (e.g., “My coach offers/gives players alternatives and options”) and socially supportive items (e.g., “My coach really appreciates players”). Responses were indicated on a five-point Likert-type scale, ranging from *Strongly disagree* (1) to *Strongly agree* (5). In previous studies, the empowering motivational scale has been found to be internally reliable (Appleton et al., 2016; Appleton et al., 2023).

To assess the satisfaction of the need for autonomy, we used the Mexican version (López-Walle et al., 2012) of the 10-item Perceived Autonomy in Sport Scale (PASS; Reinboth and Duda, 2006). An example item is “I can give my opinion.” The responses were rated on a five-point Likert-type scale ranging from (1) *Strongly disagree* to (5) *Strongly agree*. The scale has shown satisfactory reliability in previous studies (López-Walle et al., 2012).

To assess the satisfaction of the need for competence, we used the Mexican version (López-Walle et al., 2012) of the 5-items Perceived Competence Scale of the Intrinsic Motivation Inventory (IMI; McAuley et al., 1989). An example item is “I am quite skilled in my sport.” The responses were rated on a five-point Likert-type scale ranging from (1) *Strongly disagree* to (5) *Strongly agree*. The reliability of the scale has been supported in previous studies with young Mexican athletes (López-Walle et al., 2012).

To assess the satisfaction of the need for relatedness, we used the Mexican version (López-Walle et al., 2012) of the 5-item Acceptance subscale of the Perceived Relatedness Scale (PRS; Richer and Vallerand, 1998). An example item is “I feel supported.” The responses were rated on a five-point Likert-type scale ranging from (1) *Totally disagree* to (5) *Totally agree*. Previous studies have reported adequate reliability of the scale in the case of young Mexican athletes (López-Walle et al., 2012).

To assess team cohesion, we used the Spanish version (Castillo et al., 2016) of the Youth Sport Environment Questionnaire (YSEQ; Eys et al., 2009). The scale is made up of 18 items measuring two first-order factors: task cohesion with 9 items (e.g., “We have all been equally committed to the team’s objectives”) and social cohesion with 9 items (e.g., “Some of my best friends are in this team”). The responses were rated on a five-point Likert scale ranging from *Strongly disagree* (1) to *Strongly agree* (5). Cronbach’s alpha ranging from 0.82 to 0.78 were exhibited for the task and social cohesion subscales, respectively, in previous studies (Castillo et al., 2016).

### 2.5 Statistical analyses

First, validity and reliability analyses were carried out for each of the scales used. We conducted confirmatory factor analysis (CFA) to provide evidence of validity of the internal structure of the various scales in the sample. Cronbach’s alpha value was estimated for each scale as a measure of internal consistency. Second, descriptive statistics (mean and standard deviation) and bivariate correlations among the variables assessed at the two time points in the season were obtained.

Paired-samples *t*-tests were conducted to assess pre–post changes in perceptions of the motivational climate, need satisfaction, and cohesion. This pre-post comparison was carried out following a per-protocol approach, analyzing data from participants who completed assessments at both time points. This analytic strategy was adopted due to the exploratory nature of the study and the relatively small sample size, which limited the feasibility of applying more complex longitudinal models at this stage. Effect sizes (Cohen’s *d*) were calculated and interpreted according to the empirically derived benchmarks proposed by Lovakov and Agadullina (2021), where d values of 0.20, 0.60, and 0.80 indicate small, moderate, and large effects, respectively. Statistical significance was determined using a conventional two-tailed *p*-value threshold of 0.05. Confidence intervals (95%) were also calculated to provide additional information regarding the precision of the estimates. All analyses were conducted using IBM SPSS Statistics 24.

Finally, to test our hypotheses regarding the hypothesized process model, structural equation modeling (SEM) analysis with Mplus 6, using ML as method of estimation, was carried out.

Considering the sample size and the number of parameters to be estimated, we modeled relationships among observed (scale-scores) variables to ensure model stability and avoid overparameterization. The SEM analysis complements the preliminary paired-samples comparisons by allowing simultaneous estimation of the hypothesized mediational pathways. Model fit was assessed using the chi-square statistic, the root-mean-square error of approximation (RMSEA), the comparative fit index (CFI), the Tucker–Lewis index (TLI), and the standardized root-mean-square residual (SRMR). RMSEA values of about 0.08 or less would indicate a fair fit of the model, and values higher than 0.10 would indicate poor fit. CFI and TLI values above 0.90 are adopted as an indication of adequate model fit. SRMR values lower than 0.08 are generally considered a good fit. To test the indirect effects involved in the proposed mediation model, we used the bias-corrected (BC) bootstrap confidence interval method as implemented in Mplus (Lau and Cheung, 2012). If the confidence interval does not include zero, the null hypothesis of no mediation is rejected, providing empirical support for the indirect effect.

## 3 Results

### 3.1 Confirmatory factor analysis

The results of the confirmatory factor analyses were adequate, providing evidence of validity of the internal structure of the scales employed. It was observed that the *χ2/gl* were within a range of 1.90 (BPN Satisfaction at Time 2) and 2.48 (cohesion at Time 2), the CFI values were between 0.84 (empowering at Time 1) and 0.93 (BPN satisfaction at Time 2), the TLI values were between 0.83 (empowering Time 1) and 0.92 (satisfaction of the BPN at Time 2), the RMSEA is between 0.06 (empowering and satisfaction of the BPN at Time 2) and 0.09 (cohesion at Time 1), finally the SRMR is between 0.05 (satisfaction of the BPN at Time 2) and 0.09 (empowering at Time 1). These results indicate acceptable model fit. However, the CFI value of 0.84 for one of the scales approaches the lower boundary of acceptable fit and should be interpreted with caution. Detailed values are presented in Table 2.

**Table 2:** Confirmatory factor analysis.

	Time 1/Time 2				
	*χ*^2^/df	CFI	TLI	RMSEA	SRMR
M1. Empowering climate	1.93/2.06	0.84/0.86	0.83/0.85	0.08/0.06	0.09/0.08
M2. Basic psychological need satisfaction	1.91/1.90	0.87/0.93	0.86/0.92	0.08/0.06	0.08/0.05
M3. Cohesion	2.13/2.48	0.90/0.90	0.89/0.89	0.09/0.08	0.06/0.06

M1: Second-order factor model for Empowering climate; M2: Three-factor model for Satisfaction of the Basic Psychological Needs (autonomy, competence, relatedness); M3: Two-factor model for cohesion (task cohesion and social cohesion).

### 3.2 Reliability, descriptive statistics, and inter-correlations

The observed internal reliability for each of the scales was satisfactory. Cronbach’s Alpha values ranged from 0.81 (for social cohesion at Time 2) to 0.96 (for empowering climate at Time 1; Table 3). The descriptive statistics (mean and standard deviation) for each of the targeted psychological variables are shown in Table 3.

**Table 3:** Reliability and descriptive statistics at Time 1 and Time 2.

Psychological Variables	Time 1			Time 2			*t*	*d*
	*α*	M	SD	*α*	M	SD		
Empowering climate	0.96	3.83	0.97	*0.95*	*3.96*	*0.81*	−2.03*	0.34
Autonomy satisfaction	0.92	3.76	0.98	0.93	3.98	0.92	−2.71**	0.45
Competence satisfaction	0.90	4.03	0.83	0.90	4.02	0.78	0.06	0.01
Relatedness satisfaction	0.94	4.06	1.05	0.94	4.28	0.85	−2.42**	0.41
Task cohesion	0.85	3.73	0.78	0.83	3.75	0.7	−0.33	0.06
Social cohesion	0.84	3.72	0.72	0.81	3.82	0.66	−1.63*	0.27

*a* = alpha *t* = t-test *d* = Cohen’s d. **p < 0.01, *p < 0.05.

Paired-sample *t*-tests showed significant increases in athletes’ perceptions of empowering climate, autonomy satisfaction, relatedness satisfaction, and social cohesion. Effect sizes for these outcomes were small-to-moderate (*d* = 0.27–0.45), consistent with the intervention’s focus on autonomy- and socially supportive environments. By contrast, competence satisfaction and task cohesion did not change significantly (Table 3).

Regarding the associations between the psychological variables, significant and positive relationships were found between all the variables at Time 1 and Time 2 (see Table 4). A perceived empowering climate was positively related with the satisfaction of the BPNs (autonomy, competence, relatedness) and cohesion (task and social cohesion).

**Table 4:** Correlations between the psychological variables.

Psychological Variables	1	2	3	4	5	6
1. Empowering climate	–	0.75**	0.25**	0.77**	0.62**	0.45**
2. Autonomy satisfaction	0.68**	–	0.35*	0.69**	0.47**	0.39**
3. Competence satisfaction	0.22**	0.41**	–	0.38**	0.50**	0.41**
4. Relatedness satisfaction	0.64**	0.65**	0.32**	–	0.59**	0.51**
5. Task cohesion	0.67**	0.58**	0.32**	0.53**	–	0.77**
6. Social cohesion	0.40**	0.45**	0.26**	0.25**	0.60**	–

**p < 0.01, *p < 0.05. Above diagonal Time 1, below diagonal Time 2.

### 3.3 Intervention effects on the perceived empowering climate and cohesion

As shown in Table 3, perceptions of empowering climate, autonomy satisfaction, relatedness satisfaction, and social cohesion increased significantly from T1 to T2. No significant changes were observed for competence satisfaction and task cohesion. According to the benchmarks proposed by Lovakov and Agadullina (2021), the intervention produced moderate effects on autonomy satisfaction (*d* = 0.45) and relatedness satisfaction (*d* = 0.41), and a small-to-moderate effect on empowering climate perceptions (*d* = 0.34). The effect on social cohesion was small (*d* = 0.27), while changes in competence satisfaction (*d* = 0.01) and task cohesion (*d* = 0.06) were negligible. The results are consistent with the intervention’s focus on fostering autonomy-supportive and socially supportive environments.

### 3.4 Testing the hypothesized process model

**Figure 2:**
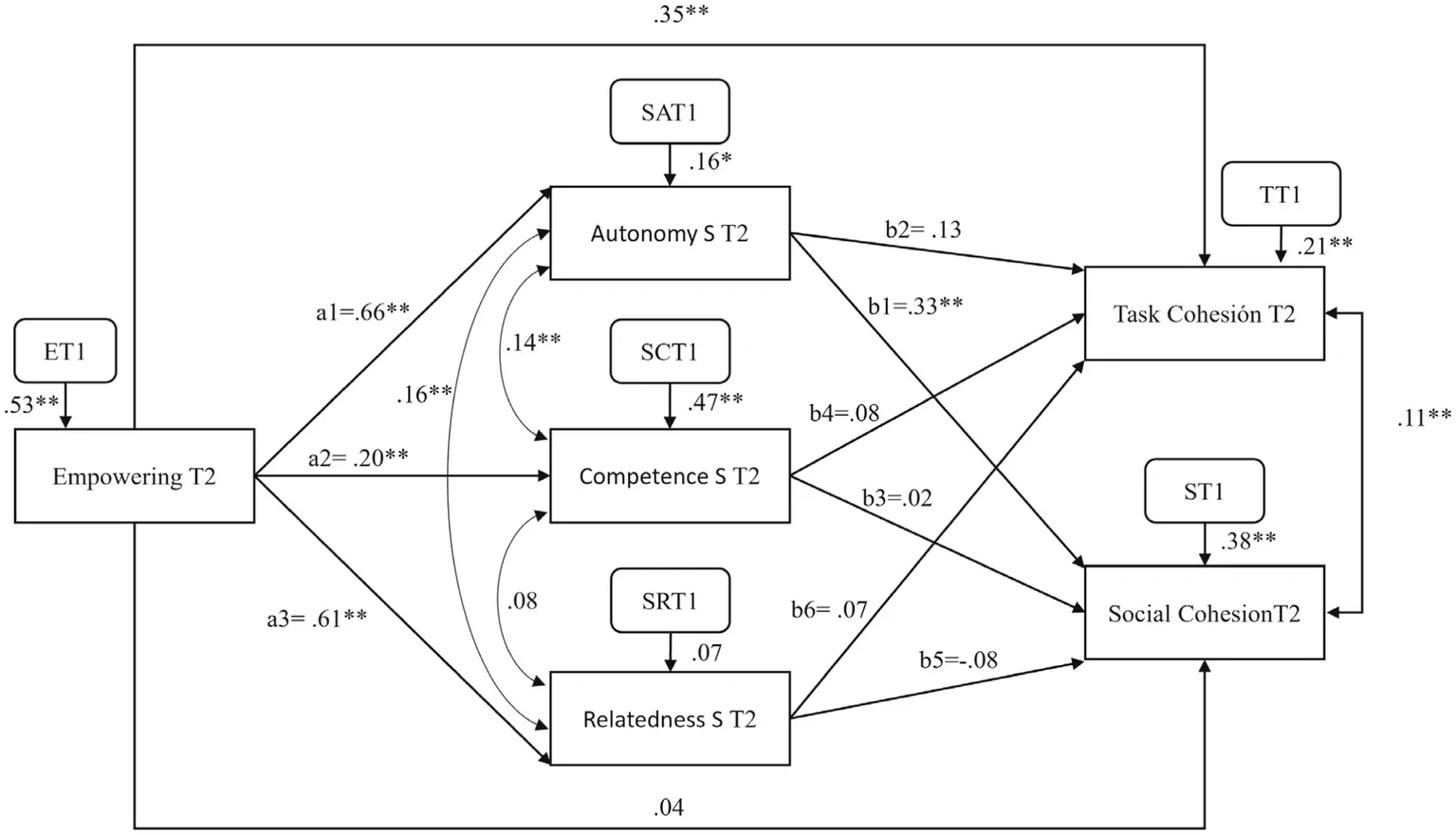
Structured equation model. ET1 = empowering at time 1; SAT1 = autonomy satisfaction at Time 1; SCT1 = competence satisfaction at time 1; SRTI = relatedness satisfaction at Time 1; TT1 = task cohesion at time 1; ST1 = social cohesion at time 1; T2 = Time 2.

The results of the structural equation modeling can be seen in Figure 2. The hypothesized model showed acceptable goodness of fit indices (*χ^2^* = 59.81, *df* = 33, CFI = 0.95, TLI = 0.90, RMSEA = 0.07, SRMR = 0.04). Changes in empowering climate positively predicted changes in autonomy satisfaction (a1 = 0.66, *p* < 0.01), competence satisfaction (a2 = 0.20, *p* < 0.01), and relatedness satisfaction (a3 = 0.61, *p* < 0.01). Likewise, changes in autonomy satisfaction positively predicted changes in social cohesion (b1 = 0.33, *p* < 0.01). Moreover, the bias-corrected (BC) bootstrap confidence interval for the estimated indirect effects (see Table 5), showed one significant indirect effect; that is, the indirect effect of empowering climate on social cohesion through autonomy satisfaction did not include zero (a1*b1 = 0.21, 95% BC bootstrap CI = [0.09, 0.35]). This finding confirms the mediating role of autonomy satisfaction in the relationship between perceptions of an empowering climate and social cohesion (Table 5).

**Table 5:** Confidence intervals for the indirect effects.

Psychological Variables	Indirect effect		
	95% lower limit	Estimate	95% upper limit
Emp - Auto S - Task C	−0.02	0.08	0.20
Emp - Auto S - Social C	0.09	0.21	0.35
Emp - Comp S - Task C	−0.08	0.02	0.05
Emp - Comp S - Social C	−0.03	0.01	0.02
Emp - Rel S - Task C	−0.04	0.04	0.23
Emp - Rel S - Social C	−0.13	−0.05	0.04

Emp = empowering, Auto S = autonomy satisfaction, Comp S = competence satisfaction, Rel S = relatedness satisfaction, Task C = task cohesion, Social C = social cohesion.

In summary, Hypothesis 1 was supported: the empowering climate significantly predicted changes in autonomy satisfaction (*β* = 0.66, *p* < 0.01), competence satisfaction (β = 0.20, *p* < 0.01), and relatedness satisfaction (β = 0.61, *p* < 0.01). Hypothesis 2 was partially supported, as only autonomy satisfaction significantly predicted changes in social cohesion (β = 0.33, *p* < 0.01). Hypothesis 3 received partial support as well: only one indirect path (empowering climate → autonomy satisfaction → social cohesion) reached statistical significance (a1*b1 = 0.21, 95% BC bootstrap CI = [0.09, 0.35]). Lastly, Hypothesis 4 was partially confirmed: there was a direct effect from empowering climate to task cohesion, whereas the direct path to social cohesion was not significant.

## 4 Discussion

Past work has pointed to the importance of the motivational climates created by coaches on the quality of the sport experience of athletes, both as individuals and also considering their views on how the team is functioning (Balaguer et al., 2015; Eys et al., 2013). The main objective of this study was to examine the effects of an extended, collaborative, multi-activity educational intervention based on the philosophy, content, and methodology embedded in the *Empowering Coaching*™ training program (Duda, 2013). The training program was delivered over the course of a sport season to coaches from a private international school. The focus of the training was to help coaches understand the importance of empowering strategies—both for the quality of their young athletes’ sport engagement and for team functioning—as well as to develop and implement strategies for creating more empowering climates.

Athletes’ perceptions of the empowering features of the coach-created climate, satisfaction of their three BPNs, and ratings of task and social cohesion were assessed before coaches commenced the training program, and at the end of the season. Overall, the results supported the predicted positive effects of the intervention. A significant increase in perceptions of the empowering features of the motivational climate was observed, reflecting a medium-to-low effect size (Table 3). There was support for the expected effects of the training program on BPN satisfaction: a significant increase in autonomy, competence and relatedness satisfaction was found. Similarly, social cohesion increased significantly from Time 1 to Time 2, whereas task cohesion remained unchanged.

Our results suggest that the creation of an empowering climate by the coaches (effects of the training program) was primarily associated with the social aspects of how team members connect with each other, while showing weaker associations with task-related facets of cohesion. Moreover, efforts to foster a more empowering climate were not significantly associated with athletes’ perceptions of competence. Rather, in this study, the *Empowering Coaching*™ program was more strongly associated with athletes’ feelings of autonomy and relatedness, suggesting a potential enhancement in their sense of having a voice on and belonging on their team as well as the quality of social interactions within the team.

Taken together, these results provide empirical support for the positive effects of the *Empowering Coaching*™ program over the course of the 9-month intervention period. Although the T2 assessment was conducted after 7 months, this timing was strategically planned to allow the integration of athletes’ feedback into the subsequent training sessions delivered during the final 2 months of the intervention. This approach enabled coaches to reflect on athletes’ perceptions and further consolidate the application of empowering strategies.

The present findings suggest that the program’s content and pedagogical approach successfully facilitated coaches’ ability to adopt and implement behaviors that promote an empowering motivational climate. Within such a climate, athletes are more likely to perceive that their evaluation is based on effort, personal improvement, and collaborative engagement rather than an emphasis on social comparison; they feel empowered to express their views and participate in decision-making processes; and they experience their coach as supportive and respectful. While cultural factors may influence how athletes perceive and respond to empowering coaching, caution is needed in attributing null or partial effects to cultural explanations without direct evidence. It could be the case that contextual considerations—such as the developmental stage of athletes, the recreational/competitive orientation of the school program, and institutional constraints on coaching practices—played a more immediate role in shaping the observed outcomes (Keegan et al., 2014; Smoll and Smith, 2006; Weiss et al., 2021).

This study also tested a hypothesized process model exploring whether changes in cohesion covaried with hypothesized antecedents, namely, athletes’ perceptions of empowering climate and BPN satisfaction. We also tested the theoretically assumed mediating role of BPN satisfaction (autonomy, competence, and relatedness) in the relationships between perceived empowering climate and both types of cohesion, as well as the direct effects of empowering climate on cohesion.

Aligned with theoretical predictions and previous research findings (Castillo-Jiménez et al., 2022; Chu et al., 2020; Ramírez, 2020), changes in the young athletes’ perceptions of the empowering climate were expected to positively predict changes in BPN satisfaction. The results provided support for this hypothesis: perceived empowering climate significantly and positively predicted changes in autonomy, competence and relatedness satisfaction. These results are aligned with other studies (Castillo-Jiménez et al., 2022; Ramírez, 2020), which from a cross-sectional perspective, found significant positive relationships between an empowering motivational climate and satisfaction of the three BPNs.

It was also hypothesized that changes in BPN satisfaction would positively predict changes in task and social cohesion. However, only autonomy satisfaction significantly predicted changes in social cohesion. These results contrast with those from Nascimento et al. (2019), where in a sample of elite futsal athletes (M age ≈ 26 years), social cohesion was positively associated with both relatedness and competence satisfaction, and task cohesion was related to all three BPN. In contrast, Erikstad et al. (2018) found, in a longitudinal study with adolescent high-performance soccer players, BPNS to be significantly associated with social cohesion, but not task cohesion—findings more consistent with the present study. Beyond developmental and cultural differences, it is also possible that the team cohesion processes in more recreational school-based settings are less directly tied to competence-related experiences compared to elite competitive contexts. Besides cultural and age-related factors, differences in the present findings may be due to the dynamic nature of cohesion and its determinants. Cohesion is traditionally defined as a dynamic construct that is affected by team performance, and other factors, and evolves over time (Carron et al., 1998).

Regarding mediation, it was hypothesized that changes in BPNS would mediate the relationship between an empowering motivational climate and cohesion. This was only supported for the autonomy → social cohesion pathway. This suggests that when coaches foster a climate that supports autonomy—by encouraging voice and choice—it enhances social bonds among teammates.

We also hypothesized direct effects of an empowering motivational climate on task and social cohesion. Results showed that empowering climate directly predicted task cohesion, but not social cohesion. While direct links between empowering climate and cohesion have not been widely studied, cross-sectional work has examined this question with respect to the sub-dimensions of empowering climates. For example, Balaguer et al. (2015) and Eys et al. (2013) reported positive associations between task-involving climates and both task and social cohesion. McLaren et al. (2015), in a longitudinal intervention study, found that a coach-initiated task-involving motivational was positively related to task cohesion, but not social cohesion—again echoing the current findings. These patterns suggest that in longitudinal designs, empowering features of the social environment may have stronger predictive power for task-oriented outcomes than for social bonds.

Several methodological limitations should be considered when interpreting these findings. First, small sample increases the risk of Type II error and may have obscured meaningful effects, particularly for more complex models (Freiman et al., 2019). The absence of a control group limits causal inference, as changes observed over time may have been influenced by extraneous contextual factors unrelated to the intervention (Shadish et al., 2002). Although repeated measures facilitate the examination of within-subject change, they do not, by themselves, warrant strong causal claims.

Building on these preliminary findings will require more rigorous designs that strengthen internal validity. Priorities include randomized controlled trials, larger samples, and intent-to-treat analyses to bolster causal inference. When randomization is not feasible, quasi-experimental designs with control or comparison groups can help isolate the effects of empowering coaching interventions from developmental or contextual influences. Longitudinal designs with multiple follow-up assessments would be valuable for evaluating the durability of effects over time and whether any observed changes are linear or curvilinear. Moreover, examining potential mediators and moderators (e.g., sport type, gender, age, motivation-related characteristics of the athletes) could clarify the mechanisms of influence and identify for whom and under what conditions empowering climates are most effective. Additionally, future studies should consider integrating multiple informants—such as coaches, parents, or independent observers—to complement athlete self-reports. Also it is important to recognize in future intervention research, that there are other motivational climates manifested in such sport settings, including the influence of the climate created by peers and parents.

In conclusion, the *Empowering Coaching*™ program as delivered in this study was found to be beneficial in increasing young athletes’ perceptions of empowering coach-created climates. Coaches who create task-involving, autonomy-supportive, and socially supportive environments not only foster stronger team cohesion but also contribute to satisfying athletes’ basic psychological needs. Overall, the findings indicate that empowering climates can enhance the quality of youth sport participation—supporting both individual development and positive team dynamics.

From an applied perspective, even small-to-moderate improvements in young athletes’ sense of autonomy, belonging, and cohesion can be meaningful in school-based sport. Nevertheless, barriers to implementation—such as time constraints, limited institutional support, and challenges in sustaining coach adherence—must be acknowledged. Addressing these barriers may require integrating shorter, more flexible training formats, ongoing supervision, or embedding *Empowering Coaching*™ principles into existing school sport curricula.

## Data Availability

The raw data supporting the conclusions of this article will be made available by the authors, without undue reservation.
